# Spatial heterogeneity in DNA methylation and chromosomal alterations in diffuse gliomas and meningiomas

**DOI:** 10.1038/s41379-022-01113-8

**Published:** 2022-06-14

**Authors:** Sandra Ferreyra Vega, Anna Wenger, Teresia Kling, Thomas Olsson Bontell, Asgeir Store Jakola, Helena Carén

**Affiliations:** 1 Department of Clinical Neuroscience, Institute of Neuroscience and Physiology, Sahlgrenska Academy, University of Gothenburg, Gothenburg, Sweden; 2 Sahlgrenska Center for Cancer Research, Department of Laboratory Medicine, Institute of Biomedicine, Sahlgrenska Academy, University of Gothenburg, Gothenburg, Sweden; 3 Department of Physiology, Institute of Neuroscience and Physiology, Sahlgrenska Academy, University of Gothenburg, Gothenburg, Sweden; 4 Department of Clinical Pathology and Cytology, Sahlgrenska University Hospital, Gothenburg, Sweden; 5 Department of Neurosurgery, Sahlgrenska University Hospital, Gothenburg, Sweden; 6 Department of Neurosurgery, St.Olavs University Hospital, Trondheim, Norway

**Keywords:** Tumour heterogeneity, DNA methylation, CNS cancer

## Abstract

Adult-type diffuse gliomas and meningiomas are the most common primary intracranial tumors of the central nervous system. DNA methylation profiling is a novel diagnostic technique increasingly used also in the clinic. Although molecular heterogeneity is well described in these tumors, DNA methylation heterogeneity is less studied. We therefore investigated the intratumor genetic and epigenetic heterogeneity in diffuse gliomas and meningiomas, with focus on potential clinical implications. We further investigated tumor purity as a source for heterogeneity in the tumors. We analyzed genome-wide DNA methylation profiles generated from 126 spatially separated tumor biopsies from 39 diffuse gliomas and meningiomas. Moreover, we evaluated five methods for measurement of tumor purity and investigated intratumor heterogeneity by assessing DNA methylation-based classification, chromosomal copy number alterations and molecular markers. Our results demonstrated homogeneous methylation-based classification of *IDH*-mutant gliomas and further corroborates subtype heterogeneity in glioblastoma *IDH*-wildtype and high-grade meningioma patients after excluding samples with low tumor purity. We detected a large number of differentially methylated CpG sites within diffuse gliomas and meningiomas, particularly in tumors of higher grades. The presence of *CDKN2A/B* homozygous deletion differed in one out of two patients with *IDH*-mutant astrocytomas, CNS WHO grade 4. We conclude that diffuse gliomas and high-grade meningiomas are characterized by intratumor heterogeneity, which should be considered in clinical diagnostics and in the assessment of methylation-based and molecular markers.

## Introduction

Diffuse gliomas and meningiomas are the most prevalent intracranial tumors of the central nervous system (CNS) in adults^1^. Meningiomas are typically benign tumors and can potentially be cured by surgical resection, whereas high-grade meningiomas carry an increased risk of recurrence and need of adjuvant treatment^2^. Diffuse gliomas, including *IDH*-mutant glioma (CNS WHO grade 2–4) and glioblastoma *IDH*-wildtype (CNS WHO grade 4), are infiltrating diseases that are currently incurable^3^. Tumor heterogeneity is considered one of the contributors to tumor recurrence and treatment failure^4^. While multiplatform high-throughput analyzes have revealed the cellular and molecular heterogeneity between and within diffuse gliomas and meningiomas^5–13^, the intratumor epigenetic heterogeneity is less investigated.

DNA methylation is a key epigenetic modification controlling several genomic functions, and methylation aberrations have been identified to play an important role in the development of human cancers^14,15^. Characterization of DNA methylation patterns has led to the identification of biologically and clinically relevant molecular subgroups of CNS tumors^5,16–19^ with diagnostic and prognostic potential. On this basis, the current 2021 WHO classification highlights the relevance of employing methylation profiling as a tool for reliable identification of tumor types and subtypes in the routine clinical practice alongside with standard methods^20^. With presumed increase in clinical use, it is necessary to establish aspects of diagnostic reproducibility, such as robustness of diagnosis throughout the tumor volume. Low level of heterogeneity would imply less risk of surgical sampling bias, a common problem in establishing WHO grade from small tissue specimens^21^ (e.g., biopsies only). In glioblastoma, WHO (2016) grade IV, we previously demonstrated that intratumor DNA methylation heterogeneity exists, with potential implications for methylation-based classification^22^.

One factor that may contribute to heterogeneity in DNA methylation profiles is cancer cells and non-cancer cell types intermingled in bulk tumor samples. The presence of normal cells within the tumors could potentially mask methylation signals from the tumor and consequently mislead biological interpretation of the methylation data^23,24^. Recently, several methods have been developed to estimate the proportion of neoplastic cells in the tumor sample, here denoted as tumor purity, based on DNA methylation array data^25–29^, but a comprehensive evaluation of these methods in different brain tumor types is limited in the literature.

In this study, we used DNA methylation data and chromosomal copy number data inferred from genome-wide DNA methylation arrays, to investigate heterogeneity across 39 diffuse gliomas and meningiomas. We further compared the performance of different methods for estimation of tumor purity to address tumor purity as a confounding factor in heterogeneity analyses. After excluding samples with low tumor purity, we found homogeneous methylation class families in *IDH*-mutant gliomas CNS WHO grade 2–4, glioblastomas *IDH*-wildtype, CNS WHO grade 4 and meningiomas, CNS WHO grade 1–2 but distinct molecular subgroups of glioblastoma *IDH*-wildtype, CNS WHO grade 4 and high-grade meningioma within some of the tumors. Differences in tumor purity could not explain the observed subtype/subclass heterogeneity in most cases, but could not be ruled out as a factor in a few. We also encountered high number of differentially methylated CpG sites within the tumors, which was associated with CNS WHO grade. Our study demonstrates intratumor heterogeneity in diffuse gliomas and meningiomas, which should be accounted for in clinical diagnostics.

## Materials and methods

### Patients and sample collection

The patients (≥18 years old) underwent surgical tumor resection of primary or recurrent diffuse lower-grade glioma (as defined by TCGA^10^) *IDH*-wildtype/*IDH*-mutant, WHO (2016) grade II–III, glioblastoma *IDH*-wildtype/*IDH*-mutant, WHO (2016) grade IV or high-grade meningioma, WHO (2016) grade II, during 2018–2020 at the neurosurgical department at the Sahlgrenska University hospital (Gothenburg, Sweden). The diagnosis of the patients followed the WHO classification criteria valid at the time of surgery (2016 WHO) and the samples were labeled according to the type of tumor; lower-grade glioma (GU-LGG-X), high-grade glioma (GU-HGG-X) and high-grade meningioma (GU-hgMNG-X), where X represents a serial ID number. Signed informed consent was obtained from all patients in this study. For each patient, three to five tumor tissue biopsies were sourced from different regions of the tumor with a spatial distance of at least 1 cm between the biopsies. Each biopsy was divided into small pieces and cryopreserved for molecular or histological assessments.

### DNA isolation and methylation array profiling

DNA extraction from fresh-frozen tumor biopsies, generation and processing of methylation data inferred from the Infinium MethylationEPIC BeadChip array (Illumina, Inc., San Diego, CA, US) as well as the analysis of differentially methylated positions (DMPs) between intratumor biopsy pairs were carried out as previously described^22^. We used the statistical software R with Rstudio (version 4.0.2) for data analysis. Prediction of the O^6^-methylguanine-DNA methyltransferase (*MGMT*) promotor methylation status^30,31^, Horvath’s methylation age^32^ and chromosomal copy number analysis were performed as previously described^22,33^. The DNA methylation-based stemness index, a predictor for oncogenic dedifferentiation, was retrieved according to the stemness index workflow from Malta et al.^34^ which scales from 0 (low stemness) to 1 (high stemness). We additionally included methylation array data from our previous study^22^ (GEO with accession GSE116298), which consisted of 58 spatially separated biopsies from 15 adult patients with a pathological diagnosis of glioblastoma *IDH*-wildtype/*IDH*-mutant, WHO (2016) grade IV and three patients diagnosed with low-grade meningioma, WHO (2016) grade I. Methylation-based diagnostic classification of the tumor biopsies was performed with the Molecular-neuropathology brain classifier version 12.5 (MNP, https://www.molecularneuropathology.org/mnp). The tumors were examined and stratified into methylation families and methylation family members (subtypes/subclasses) as indicated^35–37^ (Supplementary Table 1).

### 2021 WHO tumor reclassification

We reclassified the 2016 WHO diagnosis of the patients according to the 2021 WHO classification criteria of CNS tumors^20^ that relied on tissue-based histological data and molecular information generated at the Sahlgrenska University hospital at the time of diagnosis, and in our laboratory facility at the Sahlgrenska Center for Cancer Research. Molecular data included information on *IDH* mutation, 1p/19q codeletion, *CDKN2A/B* homozygous deletion, *EGFR* amplification, chromosome 7 gain and chromosome 10 loss. The diagnosis of astrocytoma *IDH*-wildtype, WHO (2016) grade III in patient GU-LGG-93 was changed to glioblastoma *IDH*-wildtype, CNS WHO grade 4 due to the presence of *EGFR* amplification and/or chromosome 7 and 10 alterations in the tumor. In the two glioblastomas, *IDH-*mutant, WHO (2016) grade 4 (GU-HGG-216 and GU-HGG-365), the diagnoses were updated to the newly recognized tumor type astrocytoma *IDH*-mutant, CNS WHO grade 4. In two other cases (GU-LGG-90 and GU-hgMNG-14), distinct regions of the tumors were detected, corresponding to different 2021 CNS WHO grades and therefore the overall tumor grade was based on the highest grade component present in the tumor, i.e., CNS WHO grade 4 and CNS WHO grade 2, respectively. Molecular reclassification of the diagnoses according to the 2021 WHO CNS criteria is presented in Supplementary Table 2, and it was used further for all analyses herein.

### Histology

Fresh-frozen tumor biopsies were fixated in 4% paraformaldehyde (Histolab, Sweden) overnight at 4 °C followed by dehydration in ethanol baths for 12 h and subsequently embedding in paraffin wax. 5 um of formalin-fixed and paraffin-embedded (FFPE) tumor sections were cut using a microtome (HM355S, Thermo Fisher Scientific, Waltham, MA, USA) and stained with Mayer’s hematoxylin and eosin (H&E, Histolab) for tumor purity estimations.

### Immunohistochemical staining of IDH1 and MDM2

Immunohistochemical staining of IDH1 and MDM2 was carried out on 5 um FFPE tumor tissue sections. Visualization of neoplastic cells in *IDH*-mutant gliomas was performed by immunostaining of IDH1 point mutation (R132H) on the DAKO Autostainer 48 Link and Envision FLEX detection system (Dako, Santa Clara, CA, USA). The tumor sections were dried in a heating cabinet for 1 h prior to heat-induced antigen retrieval in high pH-buffer in PT-Link (Dako). The samples were then incubated with a mouse monoclonal IDH1 antibody (DIA-H09, Dianova, Hamburg, Germany) followed by blocking with hydrogen peroxide. Horseradish peroxidase-linked secondary antibodies were added and the staining was visualized using 3,3’-Diaminobenzidine (DAB) chromogen. The sections were counterstained with Mayer’s hematoxylin.

To evaluate *MDM2* amplification in meningioma biopsies, the tumor sections underwent heat-induced antigen retrieval with citrate buffer (pH 6.0, Vector Laboratories, Burlingame, CA, USA) followed by blocking of endogenous peroxidase with 3% hydrogen peroxidase in methanol. The samples were stained with the Vectastain ABC kit (Vector Laboratories) using a mouse monoclonal MDM2 antibody (IF2; 1:800, Thermo Fisher Scientific) overnight at 4 °C according to the suppliers instructions with the addition of DAB for detection of antibody signal. The sections were then counterstained with Mayer’s hematoxylin.

### Estimation of tumor cell content

Determination of tumor purity was assessed blinded by a specialist in clinical neuropathology with FFPE H&E stained tissue sections from the fresh-frozen tumor biopsies collected in this study and from our previous study^22^. We estimated tumor purity by evaluating the fraction of neoplastic cells relative to other, nonneoplastic tissue elements such as blood vessels (white blood cells and endothelial cells), immune cells, nonneoplastic glial cells and nerve cells present in the tumor biopsies. The nonneoplastic tissue elements in the samples were considered as factors decreasing the purity of the tumors. Histopathological evaluation of tumor cell content was conducted for 123 out of 126 biopsies since there was insufficient tumor material for histology in one *IDH*-mutant glioma, CNS WHO grade 2 and two glioblastoma *IDH*-wildtype, CNS WHO grade 4 biopsies. In three other tumor biopsies (two *IDH*-mutant gliomas, CNS grade 3 and one glioblastoma *IDH*-wildtype, CNS WHO grade 4), no neoplastic cells were located by histopathological evaluation and tumor purity was set to 0%. Representative examples of estimation of tumor cell content by H&E stains are shown in Supplementary Fig. 1. In *IDH*-mutant gliomas, we further examined the reliability of histopathological tumor purity estimates by comparing these with the percentage *IDH*-mutant positive cells in the tumors determined by immunohistochemistry (Supplementary Fig. 1C, D). Tumor purity was also predicted based on the DNA methylation array data using the publicly available R packages InfiniumPurify^25,26^, PAMES^27^, and RF_purify^28^ (using the RF_absolute and RF_estimate algorithms) as described by the developers. We used methylation array data from 61 adult brain tissue samples^17^ (GSE109381) as control data set for PAMES and InfiniumPurify when estimating tumor purity in meningioma.

### Statistical analyses

All statistical analyses were conducted in R with Rstudio (version 4.0.2). Overall survival (OS) was defined as the time of diagnosis to time of death or last follow-up (June, 17th 2021). The association between OS and stemness index was predicted with the R package Survival^38^ using the Cox proportional hazards regression model test. Correlation analyses were performed using the R package corrplot^39^ and Hmisc^40^ with Pearson correlation. A correlation *p* value < 0.05 was considered statistically significant. We performed two-tailed t-test to compute the significant difference between tumor groups (*p* value < 0.05).

## Results

### Estimation of tumor purity in brain tumors

We sourced between three to five spatially separated tumor biopsies per patient diagnosed with diffuse gliomas, WHO (2016) grade II–IV or meningiomas, WHO (2016) grade II. In total, we collected 68 tumor biopsies from 21 patients and the 2016 WHO diagnoses were updated according to the 2021 WHO classification system, which resulted in 10 *IDH*-mutant gliomas (four astrocytomas, CNS WHO grade 2–4 and six 1p/19q codeleted oligodendrogliomas, CNS WHO grade 2–3), two glioblastomas *IDH*-wildtype, CNS WHO grade 4 and nine meningiomas, CNS WHO grade 2 (Supplementary Table 2). We additionally included and reclassified 58 spatially separated biopsies from our previous study^22^ for further characterization of the intratumor heterogeneity in these tumors, which included one *IDH*-mutant astrocytoma, CNS WHO grade 4, 14 glioblastomas *IDH*-wildtype, CNS WHO grade 4 and three meningiomas, CNS WHO grade 1.

**Fig. 1:**
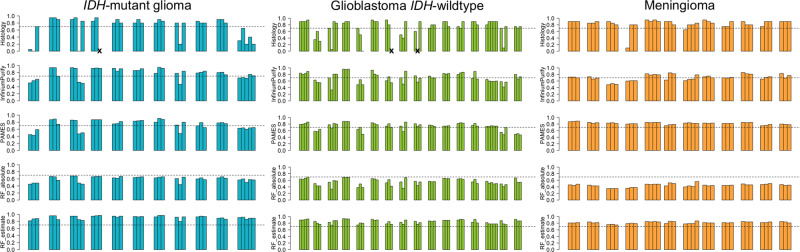
Overview of tumor purity estimations assessed by pathological evaluation based on histology, and four methylation-based methods; InfiniumPurify^25,26^, PAMES^27^, RF_absolute and RF_estimate^28^. Left; *IDH*-mutant glioma, middle; glioblastoma *IDH*-wildtype and right; meningioma. Each bar represents one biopsy and the biopsies for each patient are grouped together. Not analyzed tumor biopsies are marked with an X. The dashed line represents tumor purity of 70%.

We first evaluated the performance of five methods for estimation of tumor purity. Tumor purity was assessed based on pathological evaluation of H&E stained histological samples and calculated with InfiniumPurify, PAMES, RF_absolute and RF_estimate using methylation array data from the 126 biopsies. Tumor purity distributions for each brain tumor type is shown in Fig. 1 and Supplementary Fig. 2. As expected, due to the non-infiltrative and compact growth of meningioma, meningioma biopsies showed higher, and less variable, tumor purity than the diffuse glioma samples. For instance, the mean tumor purity based on histology in meningioma was 84% with a variance of 0.02 compared to *IDH*-mutant gliomas (mean purity 69%, variance 0.1, *p* = 9.096e−06) and glioblastoma *IDH*-wildtype (mean purity 76%, variance 0.05, *p* = 0.00836). RF_estimate predicted high levels of tumor purity in all three tumor types (median purity >80%). RF_absolute, in contrast, predicted lower tumor purities (median purity <70%) than the other methods. RF_absolute showed the highest correlation with RF_estimate (corr. = 0.89, *p* < 0.05), which is unsurprising given that they are from the same developer, but InfiniumPurify displayed overall significantly better concordance with all the other methods. Furthermore, histopathological assessment of tumor purity displayed good concordance with InfiniumPurify (corr. = 0.55, *p* < 0.05) and PAMES (corr. = 0.71, *p* < 0.05).

### Distribution of tumor purity across methylation classes

**Fig. 2:**
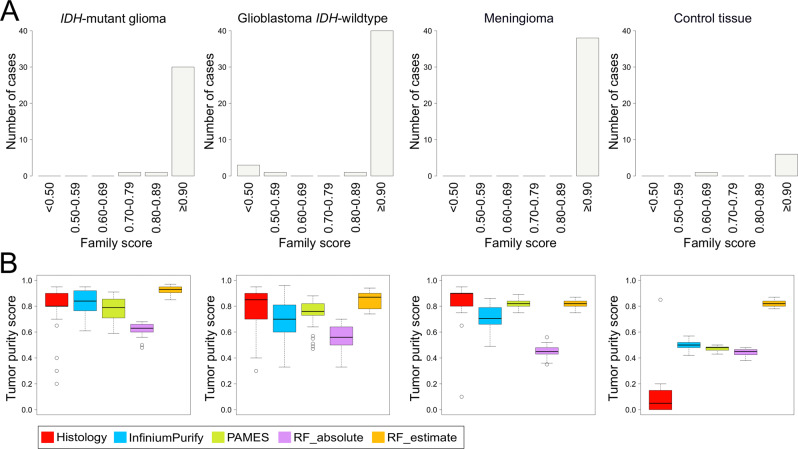
Evaluation of tumor purity across the methylation classes. All samples were classified by the MNP methylation-based brain classifier (version 12.5)^35^ and (**A**) shows the distribution of tumor biopsies by the methylation class families and class calibrated scores. **B** Tumor purity estimations among methylation classes as estimated by histology, InfiniumPurify^25,26^, PAMES^27^, RF_absolute, and RF_estimate^28^. Histopathology resulted in a more profound discrimination between the tumor methylation classes and control tissue class.

Next, we analyzed the resulting tumor purity estimates across the methylation classes predicted by the MNP brain classifier version 12.5^35^. The majority of the tumor biopsies (94%, 119/126) were classified with brain tumor methylation class families, which included diffuse glioma *IDH*-mutant (*n* = 31), glioblastoma *IDH*-wildtype (*n* = 45), meningioma (*n* = 38), low-grade ganglioglial/neuroepithelial tumor (*n* = 2) and diffuse pediatric-type high-grade glioma, H3-wildtype and *IDH*-wildtype (*n* = 3). The remaining tumor biopsies (*n* = 7) were classified as normal control tissue, an outcome also previously encountered in tumors with low tumor purity^33,36^. The vast majority of the tumor biopsies classified as *IDH*-mutant gliomas (94%, 29/31) had a calibrated family score ≥0.90 (max 1.0) but the median tumor purity differed between the methods (Fig. 2). Similarly, glioblastoma *IDH*-wildtype (89%, 40/45) had a calibrated score ≥0.90 and a slightly wider tumor purity range compared to *IDH*-mutant gliomas independent of the measurement method. Classification of meningiomas showed 100% (38/38) of the tumor biopsies with a classification score ≥0.90 and displayed less variation between the tumor purity methods. Compared with the methylation-based tumor purity calculations, the histopathological evaluation provided a more profound discrimination of tumor purity between the tumor methylation classes and the control methylation classes (*p* < 0.05).

### Diffuse gliomas and high-grade meningiomas are characterized by spatial methylation heterogeneity

The variability in cell type content within tumors has been reported as a potential source of tumor heterogeneity^23,24^ and we therefore accounted for tumor purity when investigating the intratumor heterogeneity in diffuse gliomas and meningiomas. Tumor biopsies with low tumor purity (less than 70% tumor cell content, *n* = 21) based on histopathological evaluation were excluded (Supplementary Fig. 1). We chose histopathology for tumor purity estimation since it is used in the clinic, and resulted in a more profound tumor purity discrimination between the tumor methylation class families and the control tissue class (Fig. 2). We further excluded patients with less than three intratumor biopsies and biopsies suspected as normal tissue based on flat copy number alteration (CNA) profiles (generated from the methylation array data). Furthermore, two of the three biopsies that could not be analyzed by histology, GU-HGG-204_3 and GU-HGG-224_2, were excluded due to relatively low tumor purity (mean tumor purity 60%) measured by the methylation-based tools. The third biopsy (GU-LGG-88_6) displayed high tumor purity predicted by InfiniumPurify (92%) and PAMES (87%) and was therefore retained, as these methods correlated well with histopathological evaluation (Supplementary Fig. 2).

**Fig. 3:**
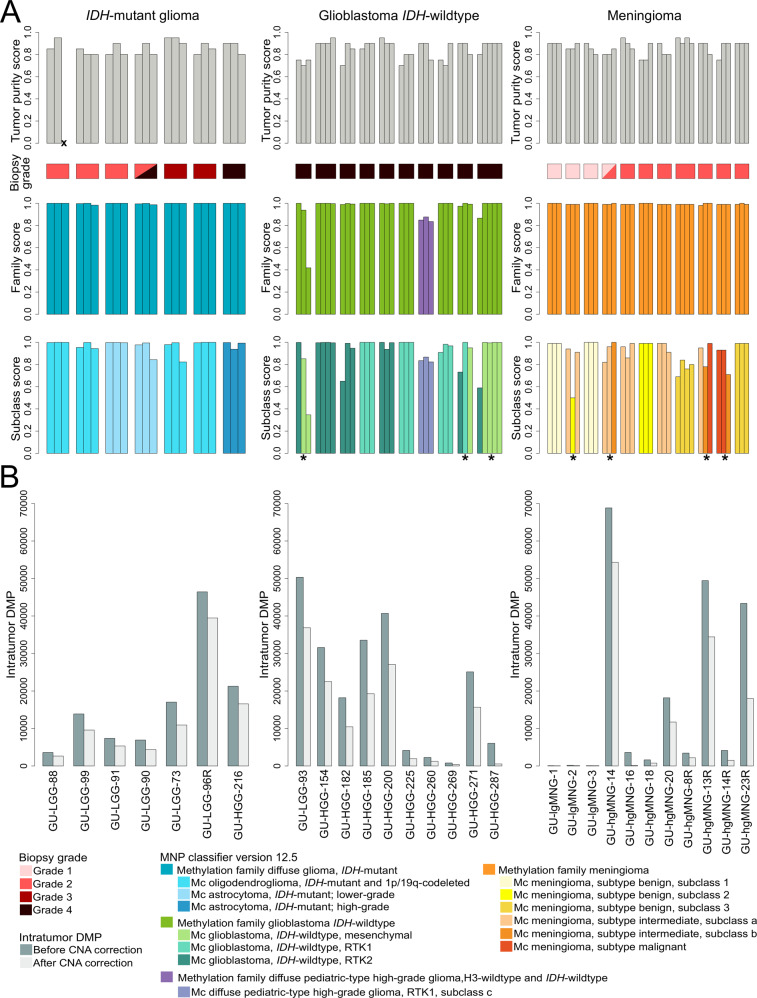
Intratumor DNA methylation heterogeneity in methylation subtyping and differentially methylated positions (DMP) between biopsy pairs. **A** Top panel: Tumor purity estimations based on histopathological evaluation. Each bar represents one biopsy and biopsies from the same patient are grouped together. X denotes a biopsy which could not be analyzed by histology. Middle panel: Biopsy grade based on 2021 WHO criteria. Bottom two panels: Methylation-based class family and subtype/subclass calibrated scores respectively from the MNP methylation-based brain classifier (version 12.5)^35^. **B** Intratumor DMPs found in the brain tumors before and after correcting for methylation artifacts of copy number alterations with SeSAMe^43^. Intratumor DMPs in glioblastoma *IDH*-wildtype from some samples and the low-grade meningioma (GU-lgMNG) are from Wenger et al.^22^ GU-LGG lower-grade glioma. GU-HGG high-grade glioma. GU-hgMNG high-grade meningioma. Recurrent tumors are denoted with an R. Patients with subtype/subclass heterogeneity are marked with an asterisk (*) symbol.

Homogeneous methylation class families and subclasses were found within all *IDH*-mutant gliomas with the MNP brain classifier (version 12.5)^35^ (Fig. 3A). Two patients (GU-LGG-73 and GU-LGG-90) showed greater differences in subclass calibrated scores within the tumors (mean difference >30%) compared to the other patients. However, the difference in tumor purity between intratumor biopsies was small (mean difference tumor purity 3%), suggesting that the calibrated scores of the classifier were not influenced by tumor purity in these cases. We previously demonstrated that multiple DNA methylation subtypes coexist within glioblastoma *IDH*-wildtype, WHO (2016) grade IV, as the tumors (e.g., GU-HGG-271) presented combinations of the RTK1/2 and mesenchymal subtypes^22^. We found two additional glioblastomas *IDH*-wildtype, CNS WHO grade 4 (GU-LGG-93 and GU-HGG-287) with subtype heterogeneity, harboring RTK2 and mesenchymal subclasses. The mean tumor purity difference between pairwise intratumor glioblastoma *IDH*-wildtype biopsies was 6% (range: 3–13%). Differences in tumor purity could thus be ruled out as the confounding factor explaining the subtype heterogeneity in GU-LGG-93 and GU-HGG-271. An overview of glioblastoma *IDH*-wildtype heterogeneity of representative cases is shown in Supplementary Fig. 3. GU-HGG-271 showed histological appearance of a glioblastoma, CNS WHO grade 4, while GU-LGG-93 showed a less cell-rich tumor. GU-LGG-93 was diagnosed as astrocytoma *IDH*-wildtype, WHO (2016) grade III but, with additional molecular data generated in this research study (*EGFR* amplification and copy number variations of chromosomes 7 and/or 10), the tumor was designated as a glioblastoma *IDH*-wildtype, CNS WHO grade 4 according to WHO 2021 criteria.

Stratification of meningioma by methylation profiling resulted in subtype heterogeneity in three out of eight high-grade meningiomas and one out of three low-grade meningiomas, as these had combinations of the benign, intermediate and/or malignant subtypes. Moreover, two high-grade meningiomas (GU-hgMNG-14 and GU-hgMNG-13R) showed subclass heterogeneity within the intermediate subtype, as the subclasses *a* and *b* were present in the tumors. The mean tumor purity difference between pairwise intratumor biopsies in high-grade meningioma was 6% (range: 0–10%), indicating that differences in tumor purity were not causing the observed subtype/subclass heterogeneity in these meningioma patients. For instance, the biopsy with the lowest tumor purity in GU-hgMNG-14R was not the one displaying a different subtype but it was one of the biopsies with as high tumor purity as another intratumor biopsy, i.e., tumor purity was not the underlying factor of the observed subtype heterogeneity. Similarly, the mean tumor purity difference in the low-grade meningioma case with subtype heterogeneity (GU-lgMNG-2) was 3%, which indicated that variation in tumor purity was not causing the observed subtype heterogeneity. An overview of meningioma heterogeneity of representative cases is shown in Supplementary Fig. 4.

Differential methylation at individual CpG sites have been associated with malignant recurrence in glioma^41^ and meningioma^42^. We therefore identified and compared the number of differentially methylated CpG sites between intratumor biopsies and examined its association to tumor heterogeneity (Fig. 3B). The mean number of DMPs within *IDH*-mutant glioma was 16,649 (max: 46,445, min: 3609) and the number of intratumor DMPs were significantly increased in *IDH*-mutant gliomas of CNS WHO grade 3–4 (mean number of intratumor DMPs: 22,918) compared to CNS WHO grade 2 tumors (mean number of intratumor DMPs: 8289). The mean number of intratumor DMPs in glioblastoma, *IDH*-wildtype was 21,279 (max: 50,324, min: 820). The mean number of intratumor DMPs was higher in high-grade meningioma (mean: 24,082, max: 68,819, min: 1632) than the previously observed mean of 110 in low-grade meningioma^22^. This suggests that more malignant tumors are more heterogeneous than tumors of lower-grades. GU-hgMNG-14, which consisted of CNS WHO grade 1 and 2 tumor components, displayed the highest number of intratumor DMPs among meningiomas, likely indicating the underlying tumor biology reflected in the varying tumor grades. We then adjusted for artifactual variation of methylation array data in regions with CNA using the processing pipeline SeSAMe^43^. The number of intratumor DMPs was slightly reduced; mean number of intratumor DMPs in *IDH*-mutant glioma: 12,704, glioblastoma *IDH*-wildtype: 13,599 and high-grade meningioma: 15,393. There was no association between the number of intratumor DMPs and the difference in tumor purity between intratumor biopsy pairs (Supplementary Fig. 5), indicating that intratumor DMPs were not affected by differences in tumor purity.

### Spatial heterogeneity in DNA methylation age, stemness index and *MGMT* promotor methylation

**Fig. 4:**
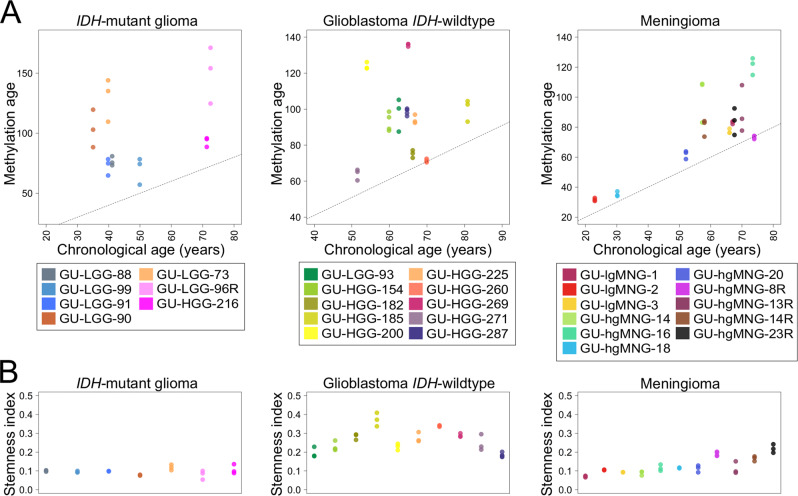
Inter- and intratumor heterogeneity in methylation-based biomarkers. **A** Methylation age varies within *IDH*-mutant glioma, glioblastoma *IDH*-wildtype and high-grade meningioma. The dashed line represents y = x as a reference. **B** Stemness index differs more in glioblastoma *IDH*-wildtype and high-grade meningioma compared to *IDH*-mutant glioma. Methylation age in glioblastoma *IDH*-wildtype from some samples and low-grade meningioma (GU-lgMNG) are from Wenger et al.^22^ GU-LGG lower-grade glioma. GU-HGG high-grade glioma. GU-hgMNG high-grade meningioma. Recurrent tumors are denoted with an R. Each biopsy is represented by a circle and color coded according to patient identity.

Epigenetic clocks have gained interest for predicting cancer risk and prognosis in adult and pediatric brain tumors and an accelerated epigenetic age has been associated with various methylation-based glioma subtypes^32,44–46^. As previously reported^22^, we detected variability in methylation age within glioblastoma *IDH*-wildtype/*IDH*-mutant, WHO (2016) grade IV, while low-grade meningioma samples exhibited a more homogeneous methylation age. We thus investigated if methylation age differed within *IDH*-mutant gliomas and high-grade meningiomas using the Horvath clock. *IDH*-mutant gliomas showed an accelerated methylation age compared to the patients’ chronological age, and methylation age varied within these tumors (mean difference methylation age: 15 years; Fig. 4A). Methylation age was also accelerated in high-grade meningiomas and methylation age differences within these tumors were higher than the previously observed in low-grade meningiomas (mean difference methylation age: 8 and 3 years respectively).

Recently, the methylation-based stemness index has been proposed as a marker of cancer progression in brain tumors including glioma^34^. We therefore examined whether there are differences in stemness index in diffuse gliomas and meningiomas, and further analyzed if stemness index correlated with prognosis in glioblastoma *IDH*-wildtype. We detected a significantly higher stemness index in glioblastoma *IDH*-wildtype compared to *IDH*-mutant glioma (mean: 0.25 and 0.10, respectively; *p* = 6.72e−16; Fig. 4B). Higher differences in stemness index were also detected within glioblastoma *IDH*-wildtype compared to *IDH*-mutant glioma (mean: 0.024 vs. 0.012, *p* = 0.056). We observed a strong correlation between stemness index and the patients’ chronological age in glioblastoma *IDH*-wildtype (Pearson’s correlation 0.67, *p* = 5.697e−05) and a negative association to overall survival (*p* = 3.650e−08). The stemness index in meningioma was also significantly higher in high-grade meningioma compared to low-grade meningioma (mean 0.15 vs. 0.10, respectively, *p* = 2.64e−05) and it varied intratumorally in one high-grade meningioma patient.

The methylation status of the *MGMT* promotor is a clinical predictive and prognostic biomarker in glioblastoma^47,48^ and has also been suggested as a prognostic biomarker in diffuse lower-grade gliomas^49^. Moreover, we previously found heterogeneous *MGMT* methylation in the only glioblastoma *IDH*-mutant, WHO (2016) grade IV patient in a cohort of 12 glioblastomas, WHO (2016) grade IV^22^. In this study, we therefore assessed *MGMT* methylation in *IDH*-mutant gliomas. *MGMT* promotor methylation status was homogeneous in *IDH*-mutant glioma CNS WHO grade 2–3 (data not shown).

### Heterogeneous copy number alterations within diffuse gliomas and high-grade meningiomas

**Fig. 5:**
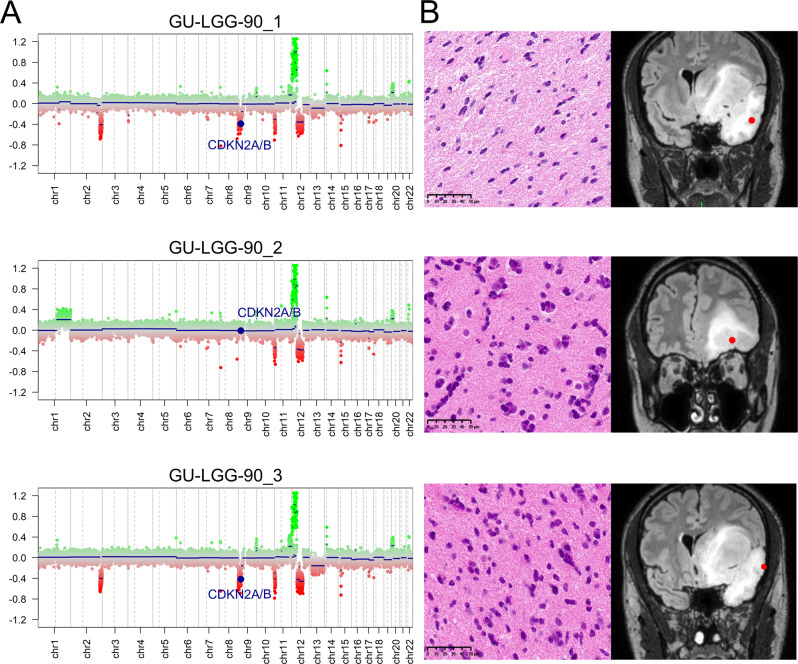
Copy number alterations inferred from methylation array data differed intratumorally in *IDH*-mutant glioma. **A** Heterogeneous status of *CDKN2A/B* homozygous deletion was detected in one out of two patients with *IDH*-mutant astrocytoma, CNS WHO grade 4 (GU-LGG-90). **B** Left panel: Hematoxylin and eosin stain and Right panel: MRI scans of the tumors for localization of the biopsies (red dot). Scale bars: 50 µm.

**Fig. 6:**
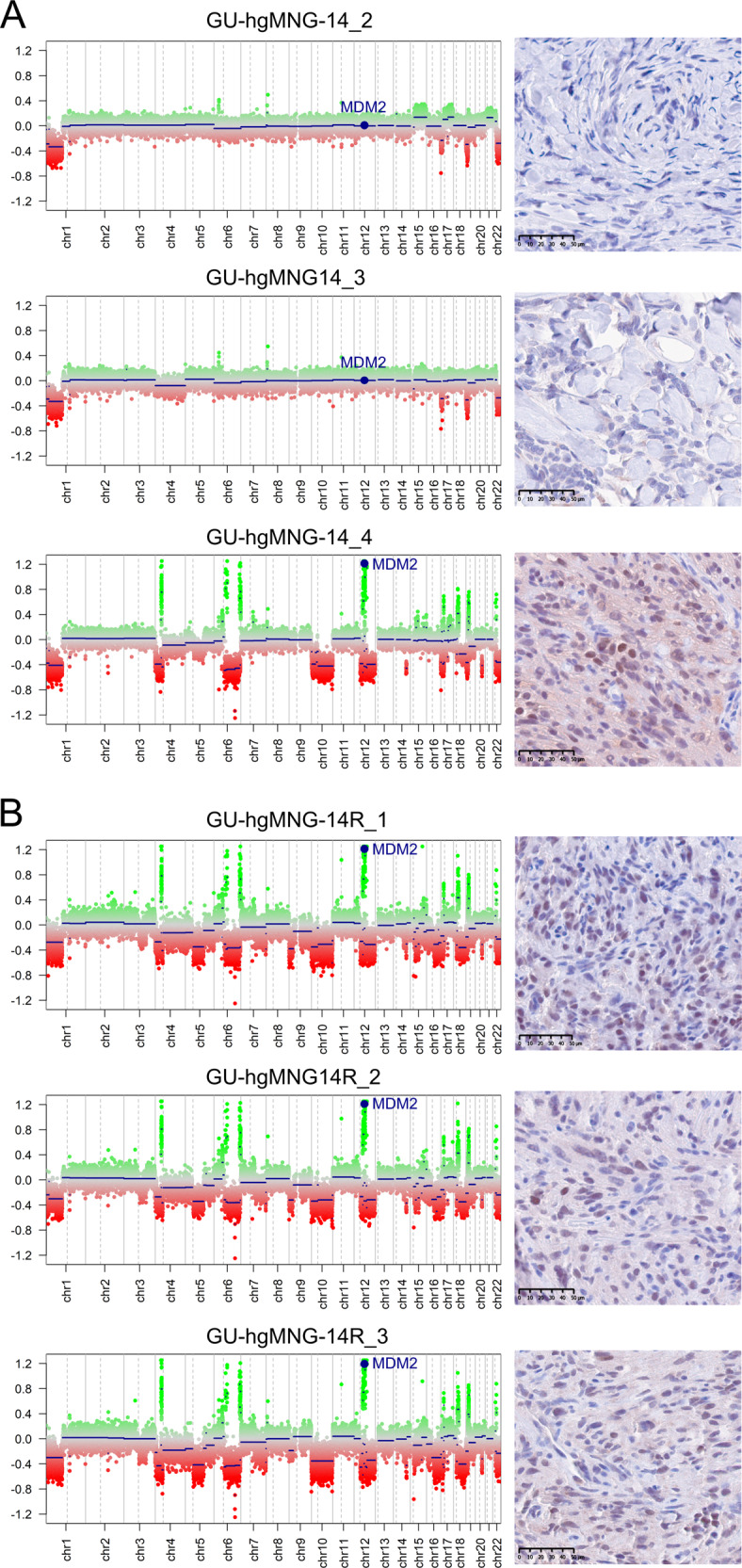
Copy number alterations inferred from methylation array data differed intratumorally in meningioma. **A** Left panels: Heterogeneous status of *MDM2* amplification was identified in the primary tumor of one out of seven high-grade meningioma patients (GU-hgMNG-14). Right panels: *MDM2* status was confirmed by immunohistochemical staining of MDM2. **B** Left panels: Amplification of *MDM2* was identified in all biopsies of the recurrent tumor (GU-hgMNG-14R). Right panels: immunohistochemical staining of MDM2. Recurrent tumors are denoted with an R. Scale bars: 50 µm.

Heterogeneous chromosomal CNA are found within tumors including glioblastoma^9^ and meningioma^7^. To explore the spatial genomic heterogeneity in our cohort of *IDH*-mutant gliomas and high-grade meningiomas, we derived CNA profiles from the methylation array data for each tumor biopsy. We detected intratumoral differences in copy number aberrations in four out of six analyzed *IDH*-mutant gliomas. In two of the patients, copy number heterogeneity in chromosome 14q was detected. Homozygous deletion of *CDKN2A/B*, which has recently been established as a biomarker of grading and prognosis in 2021 WHO *IDH*-mutant astrocytic gliomas^20^, was heterogeneous in one out of two *IDH*-mutant astrocytomas, CNS WHO grade 4 (GU-LGG-90), as two of the three biopsies presented homozygous deletion of *CDKN2A/B* (Fig. 5A). We did not detect differences in histopathological features within the tumor (Fig. 5B) but, the loss of *CDKN2A/B* in certain regions of the tumor could likely explain the rapid progression of the patient with a clinical diagnosis of diffuse astrocytoma *IDH*-mutant, WHO (2016) grade II. Other heterogeneous chromosomal aberrations in GU-LGG-90 included gain of chromosome 1q and focal deletion in chromosome 2q. Furthermore, we observed differences in CNA within three out of eight high-grade meningiomas, where the most common differences were partial or whole loss of chromosomes 4 and 6. Notably, one of the patients (GU-hgMNG-14) showed heterogeneous amplification of the proto-oncogene *MDM2*. The occurrence of *MDM2* amplification is a rare genomic alteration in atypical meningiomas associated with the malignant pathogenesis of the tumor^50,51^. In this case, the high-level amplification of *MDM2* that was found in one out of the three primary biopsies (GU-hgMNG-14_4, Fig. 6A) was also found in all the three recurrent biopsies (GU-hgMNG-14R) (Fig. 6B). Further histopathological examination of the primary tumor in GU-hgMNG-14 revealed a region of the tumor with increased cellularity and presence of larger nucleoli, which might indicate a more aggressive meningioma (GU-hgMNG-14_4) and other regions with histopathological benign phenotypes (GU-hgMNG-14_2 and 3), reflecting the observed CNA heterogeneity in the primary tumor (Supplementary Fig. 4).

## Discussion

DNA methylation profiling has gained increasing importance in the decision-making process for tumor diagnostics. Recently, the current 2021 WHO classification system for CNS tumors introduced methylation profiling as an essential diagnostic criterion for certain tumor types, or desirable in many cases, alongside with standard methods^20^. Heterogeneity in DNA methylation or in chromosomal CNAs, inferred from the methylation array, could thus potentially affect specific biomarkers. Therefore, we aimed to investigate the intratumor genetic and epigenetic heterogeneity in diffuse gliomas and meningiomas through analysis of 126 spatially separated tumor biopsies from 39 adult patients. We evaluated the impact of intratumor variability on methylation-based classification, CNAs and methylation-based biomarkers and investigated tumor purity as a confounding factor in tumor heterogeneity analyses.

We detected intratumor glioblastoma *IDH*-wildtype, CNS WHO grade 4 heterogeneity in the methylation subclasses, which is in agreement with our previous study^22^. This was also further supported by a previous study^52^, where subtype heterogeneity was found in five out of 11 glioblastomas compared to three out of 10 tumors in our study. This difference in frequency can be attributed to the distinct methodologies chosen to approach tumor purity. Intratumor subtype heterogeneity was previously detected in three out of 16 *IDH*-mutant gliomas^52^, however we found homogeneity in the methylation class families and subclasses in all *IDH*-mutant glioma analyzed. Molecular stratification of meningiomas based on DNA methylation profiles has been shown to provide a more accurate prediction of clinical behavior than the WHO classification^13,18,19^. Intratumor heterogeneity was observed in DNA methylation profiles of high-grade meningiomas^6^, and we detected multiple methylation subtypes and subclasses coexisting within high-grade meningiomas. In two of these tumors, we observed combinations of the malignant/intermediate subtypes, which could be reflected by the similar biology of these subgroups as previously identified^18^.

Prediction of age by methylation profiling has been proposed as a prognostic biomarker in diffuse gliomas^45,46^. We previously demonstrated methylation age heterogeneity within glioblastoma, *IDH*-wildtype WHO (2016) grade IV and homogeneity in low-grade meningioma^22^. We therefore calculated methylation age in the present study and found it to be heterogeneous within *IDH*-mutant gliomas and high-grade meningiomas. Similarly, the predicted stemness index significantly increased with tumor grade in our cohort, which is in agreement with Malta et al.^34^. Furthermore, we found greater variability in the stemness index between and within glioblastoma *IDH*-wildtype compared to *IDH*-mutant glioma, and also between high-grade meningioma compared to low-grade meningioma. Further validation with larger cohorts is therefore required to validate the prognostic value of these biomarkers.

The 2021 WHO criteria has recognized *CDKN2A/B* homozygous deletion as a biomarker of grading and prognosis in *IDH*-mutant astrocytic gliomas and the presence of *CDKN2A/B* homozygous deletion designates a CNS WHO grade 4 tumor^20^. We identified intratumor heterogeneity of the *CDKN2A/B* deletion in one of the two *IDH*-mutant astrocytomas, CNS WHO grade 4 in our cohort after excluding samples with low tumor purity. To the best of our knowledge, intratumor heterogeneity of *CDKN2A/B* has previously not been reported. Given that this alteration is a late event associated with malignant transformation it could potentially explain this finding. Variability in *CDKN2A/B* copy number alteration indicates that there is a risk of sampling bias that may lead to misclassification of tumor grade in *IDH*-mutant astrocytomas. This in turn may have implications on the therapeutic approach as according to guidelines, a wait-and-scan procedure is performed following a (near) radical surgery of diffuse lower-grade gliomas *IDH*-mutant^3^. Intratumor copy number heterogeneity was also observed in one high-grade meningioma affecting the *MDM2* proto-oncogene. Amplification of the *MDM2* is a rare genomic alteration in high-grade meningioma and it is considered to drive malignant progression^50,51^. In our study, the presence of *MDM2* amplification varied within the primary tumor in patient GU-hgMNG-14, but it was stable in the recurrent tumor, thus reflecting the malignant progression of the tumor. Intratumor heterogeneity of *MDM2* could potentially have implications when evaluating the clinical outcome of meningioma patients, for instance in clinical trials of targeted therapeutics with *MDM2* inhibitors. Further studies are needed to improve our understanding of *MDM2* heterogeneity and its clinical impact in meningioma patients.

Methylation values are based on the type of cells constituting the tumor tissue, including cancer cells and normal cells such as immune cells, which could contribute to tumor heterogeneity. We therefore evaluated the performance of different methods to assess tumor purity in our cohort: histopatholology, InfiniumPurify, PAMES, RF_absolute and RF_estimate. Histopathological evaluation was in good agreement with the methylation tools InfiniumPurify and PAMES and could better discriminate between tumor methylation classes from the normal tissue class than the other methods. One should note that the different computational methods are based on the same material, whereas histology was made on an adjacent tumor section from the same biopsy. In agreement with Johann et al.^28^ RF_estimate systematically predicted higher tumor purities in diffuse gliomas than the other methods and RF_absolute estimated the lowest tumor purities independently of the tumor type. This warrants some cautions when setting a general threshold when selecting samples for further analysis as it may vary depending on the method, the type of tumor, as well as the specific research question. We included biopsies with >70% tumor purity, as previously recommended^17,36^, for intratumor heterogeneity analyses. The intratumor subtype heterogeneity found in glioblastoma *IDH*-wildtype and high-grade meningioma was not driven by differing tumor content within the tumors in most of the cases. The intratumor heterogeneity in diffuse glioma and meningioma was also detected at individual CpG sites. We observed that the number of DMPs was not associated with differences in tumor purity between intratumor biopsy pairs demonstrating that variable tumor content was not causing such methylation heterogeneity in these tumors. The high intratumor DMP heterogeneity found in patient GU-hgMNG-14 could, however, be explained by the distinct phenotypes with clearly two tumor components and corresponding different CNS WHO grades of these.

In conclusion, our study demonstrates the variability in DNA methylation within diffuse gliomas and meningiomas that potentially affects tumor classification and interpretation of methylation-based biomarkers. Using chromosomal copy number profiles inferred from the array data, we also demonstrate CNA heterogeneity, with implications for clinical diagnosis and prognosis.

## Supplementary information

Supplementary Figures

Supplementary Table 1

Supplementary Table 2

## Data Availability

The datasets used and/or analyzed during the current study are available from the corresponding author on reasonable request.
