# Supplementary material for: Spatial heterogeneity in DNA methylation and chromosomal alterations in diffuse gliomas and meningiomas
Source: Mod Pathol. 2022 Jun 14;35(11):1551–61. doi: 10.1038/s41379-022-01113-8 (PMC9596370; doi:10.1038/s41379-022-01113-8)

Spatial heterogeneity in DNA methylation and chromosomal alterations in diffuse gliomas and meningiomas

Sandra Ferreyra Vega[1,2], Anna Wenger[2], Teresia Kling[2], Thomas Olsson Bontell[3,4], Asgeir Store Jakola[1,5,6] and Helena Carén[2]

Authors Affiliations

1. Department of Clinical Neuroscience, Institute of Neuroscience and Physiology, Sahlgrenska Academy, University of Gothenburg, Gothenburg, Sweden
2. Sahlgrenska Center for Cancer Research, Department of Laboratory Medicine, Institute of Biomedicine, Sahlgrenska Academy, University of Gothenburg, Gothenburg, Sweden
3. Department of Physiology, Institute of Neuroscience and Physiology, Sahlgrenska Academy, University of Gothenburg, Gothenburg, Sweden
4. Department of Clinical Pathology and Cytology, Sahlgrenska University Hospital, Gothenburg, Sweden
5. Department of Neurosurgery, Sahlgrenska University Hospital, Gothenburg, Sweden
6. Department of Neurosurgery, St.Olavs University Hospital, Trondheim, Norway

Corresponding author: Helena Carén, Associate Professor, PhD, Sahlgrenska Center for Cancer Research, Medicinaregatan 1F, 405 30 Gothenburg, Sweden (+46 (0)31 786 3838, helena.caren@gu.se)

SUPPLEMENTARY FIGURE CAPTIONS

Supplementary Figure 1. Representative hematoxylin and eosin (H&E) and immunohistochemistry (IHC) images showing high or low tumor purity estimates of the research biopsies sampled from A. glioblastoma *IDH*-wildtype, B. meningioma and C. *IDH*-mutant glioma tumors. Please note that diagnostic meningioma samples rarely show low tumor cell content and the low tumor purity defined for meningioma in this particular biopsy reflects a small tumor component and a large adjacent non-cancer tissue D. The percentage of IDH1 (R132H) positive cells in *IDH*-mutant gliomas estimated by IHC significantly correlated with histopathological evaluation of tumor purity. Scale bars: 100µm. The dashed line represents y=x as a reference. Regression line is colored in red.

Supplementary Figure 2. Evaluation of tumor purity in brain tumors. Top panel: Distribution of tumor purity estimations based on histopathology, InfiniumPurify[1,2], PAMES[3], RF_absolute and RF_estimate[4] for *IDH*-mutant glioma, glioblastoma *IDH*-wildtype and meningioma. Bottom panel: Correlation between the methods for estimation of tumor purity in the brain tumors. The dashed line represents y=x as a reference. Regression lines are color coded according to the color of each tumor type.

Supplementary Figure 3. Methylation-based classifications and calibrated scores together with representative hematoxylin and eosin images and MRI scans of glioblastoma *IDH*-wildtype cases showing intratumor methylation subclass heterogeneity; A. Case GU-LGG-93 and B. Case GU-HGG-271. Red dots: Location of the biopsy in the tumor. Scale bars: 50µm.

Supplementary Figure 4. Methylation-based classifications and calibrated scores together with representative hematoxylin and eosin images and MRI scans of meningioma cases showing intratumor methylation subclass heterogeneity; A. Case GU-hgMNG-14 and B. Case GU-hgMNG-14R. Recurrent tumors are denoted with an R. Red dots: Location of the biopsy in the tumor. Scale bars: 50µm.

Supplementary Figure 5. The number of differentially methylated positions (DMP) between intratumor biopsies was not associated with differences in tumor purity estimated by histopathological evaluation. Regression lines are colored in red.

REFERENCES

1. Zheng X, Zhang N, Wu HJ, Wu H. Estimating and accounting for tumor purity in the analysis of DNA methylation data from cancer studies. Genome biology. 2017; 18(1):17.
2. Qin Y, Feng H, Chen M, Wu H, Zheng X. InfiniumPurify: An R package for estimating and accounting for tumor purity in cancer methylation research. Genes & diseases. 2018;5(1):43-5
3. Benelli M, Romagnoli D, Demichelis F. Tumor purity quantification by clonal DNA methylation signatures. Bioinformatics (Oxford, England). 2018;34(10):1642-9.

4. Johann PD, Jager N, Pfister SM, Sill M. RF_Purify: a novel tool for comprehensive analysis of tumor-purity in methylation array data based on random forest regression. BMC Bioinformatics. 2019;20(1):428.

Supplementary Figure 1.

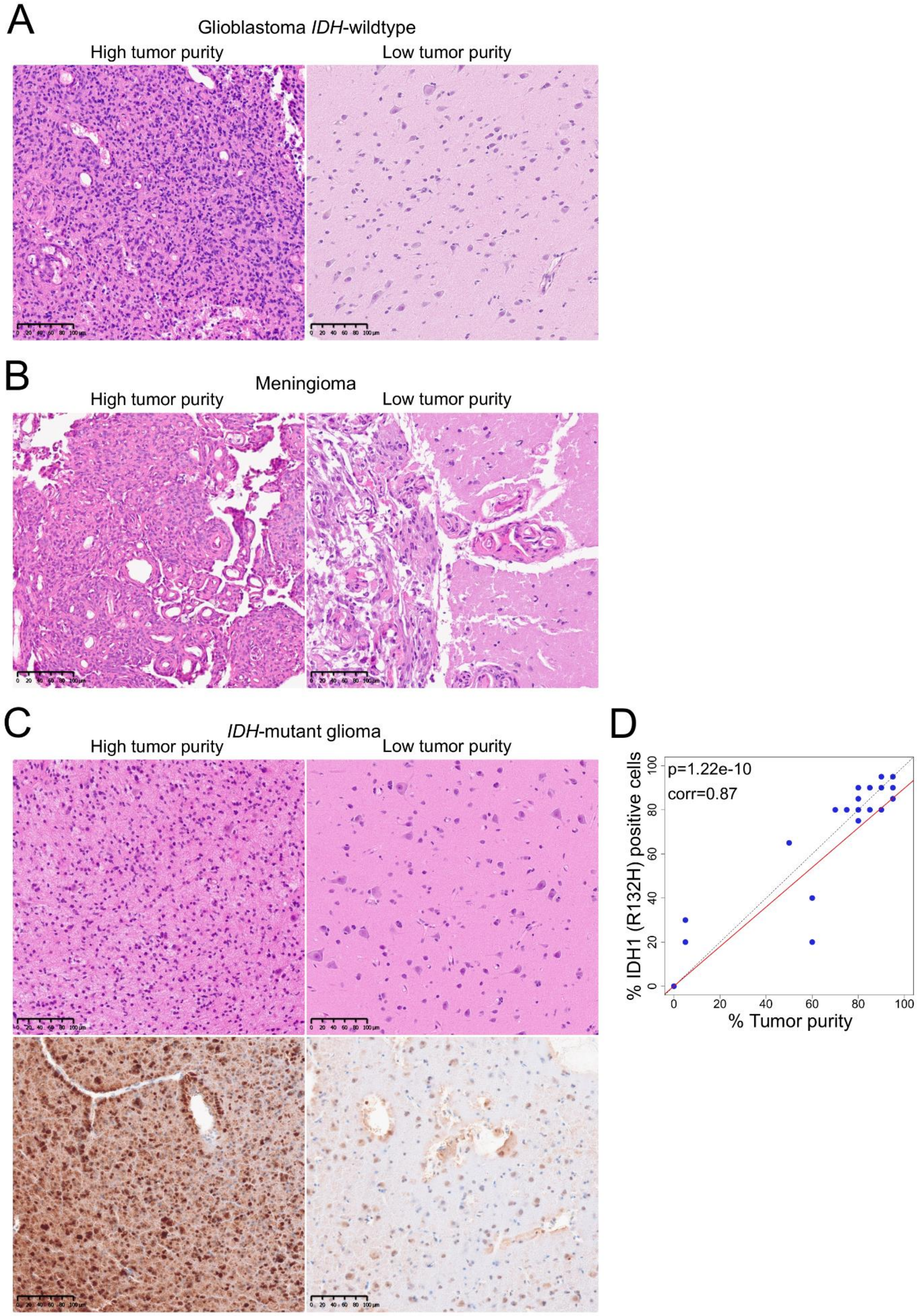

Supplementary Figure 2.

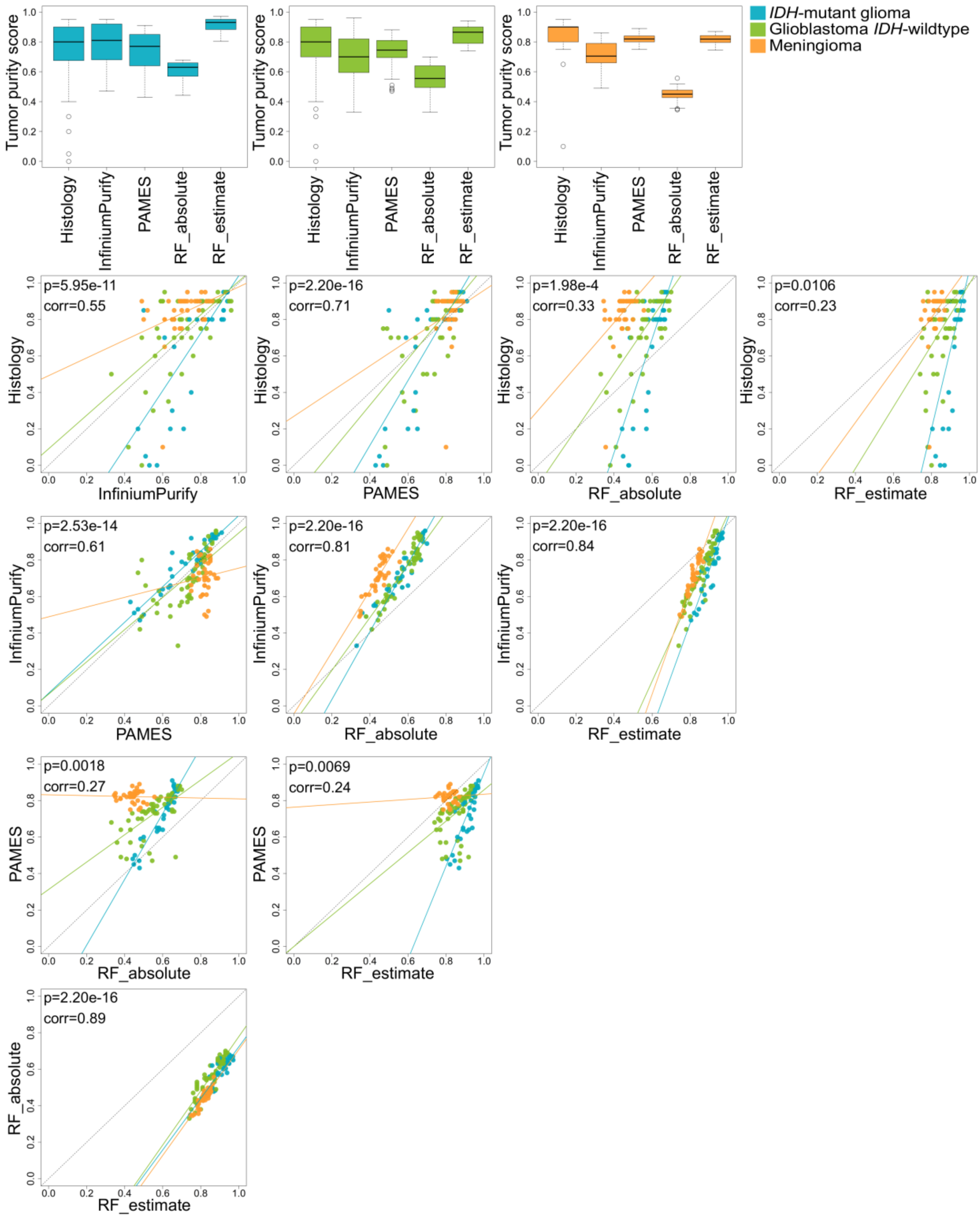

Supplementary Figure 3.

A

GU-LGG-93_1

Glioblastoma *IDH*-wildtype: 0.99972,

MC glioblastoma *IDH*-wildtype, RTK2 subtype: 0.99937

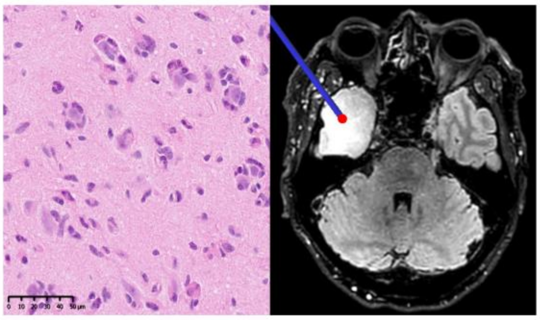

GU-LGG-93_2

Glioblastoma *IDH*-wildtype: 0.93845,

MC glioblastoma, *IDH*-wildtype, mesenchymal subtype: 0.85146

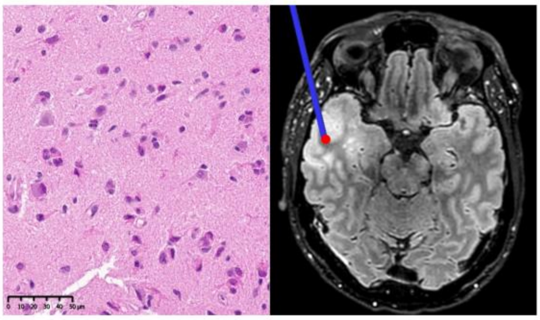

GU-LGG-93_3

Glioblastoma *IDH*-wildtype: 0.41849,

MC glioblastoma, *IDH*-wildtype, mesenchymal subtype: 0.34668

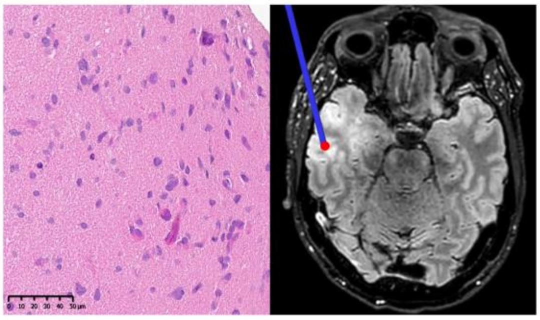

B

GU-HGG-271_1

Glioblastoma *IDH*-wildtype: 0.97368,

MC glioblastoma *IDH*-wildtype, RTK2 subtype: 0.73215

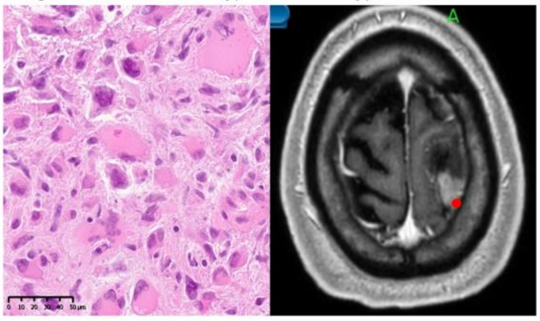

GU-HGG-271_2

Glioblastoma *IDH*-wildtype: 0.99994,

MC glioblastoma, *IDH*-wildtype, RTK1 subtype: 0.99971

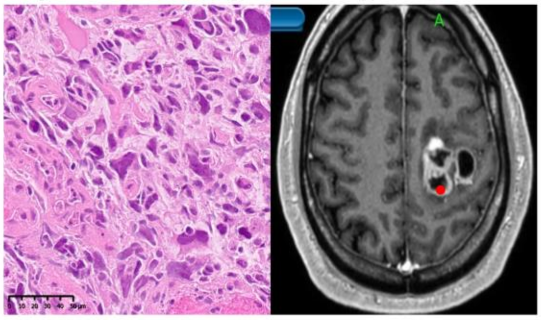

GU-HGG-271_3

Glioblastoma *IDH*-wildtype: 0.99194,

MC glioblastoma, *IDH*-wildtype, mesenchymal subtype: 0.95047

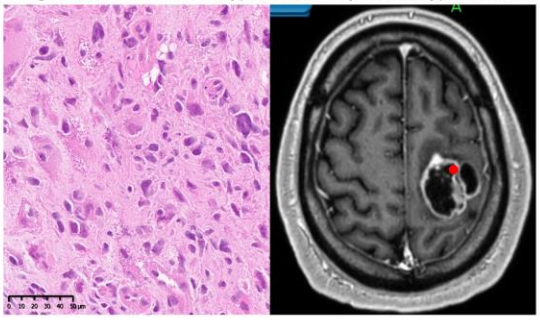

Supplementary Figure 4.

A

GU-hgMNG-14_2

Meningioma: 0.99876,

MC meningioma, subtype intermediate, subclas a: 0.81496

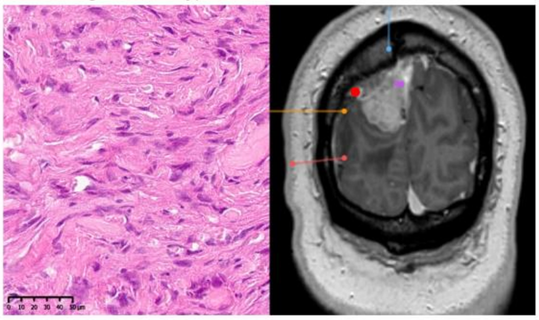

GU-hgMNG-14_3

Meningioma: 0.99903,

MC meningioma, subtype intermediate, subclas a: 0.96410

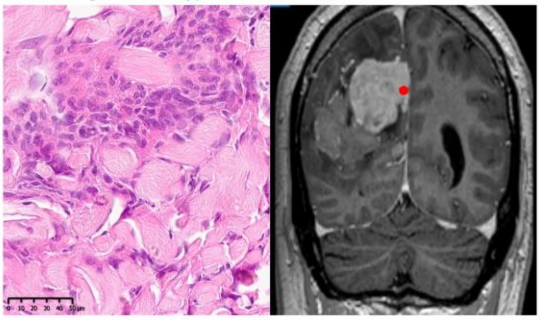

GU-hgMNG-14_4

Meningioma: 0.99999,

MC meningioma, subtype intermediate, subclas b: 0.9998

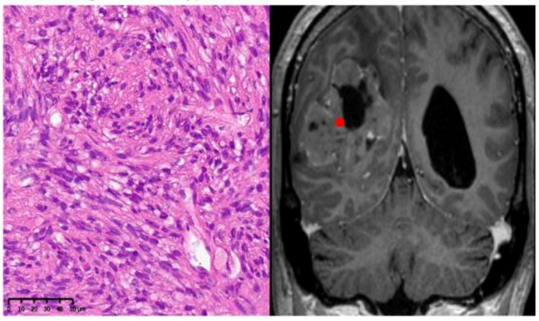

B

GU-hgMNG-14R_1

Meningioma: 0.99628,

MC meningioma, subtype malignant: 0.93406

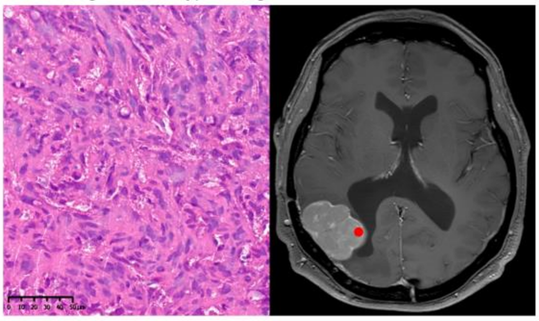

GU-hgMNG-14R_2

Meningioma: 0.99756,

MC meningioma, subtype malignant: 0.993409

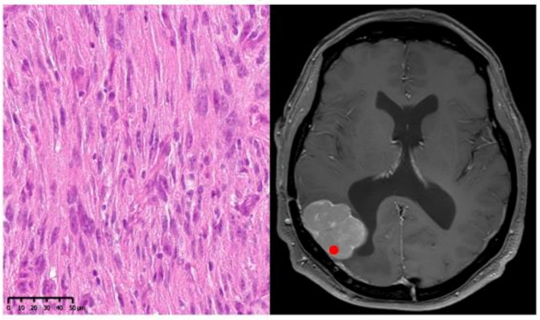

GU-hgMNG-14R_3

Meningioma: 0.99735,

MC meningioma, subtype intermediate, subclas b: 0.70575

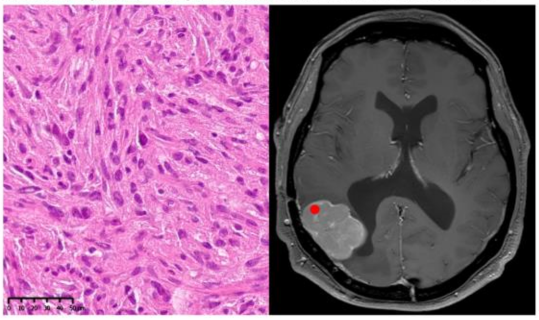

Supplementary Figure 5.

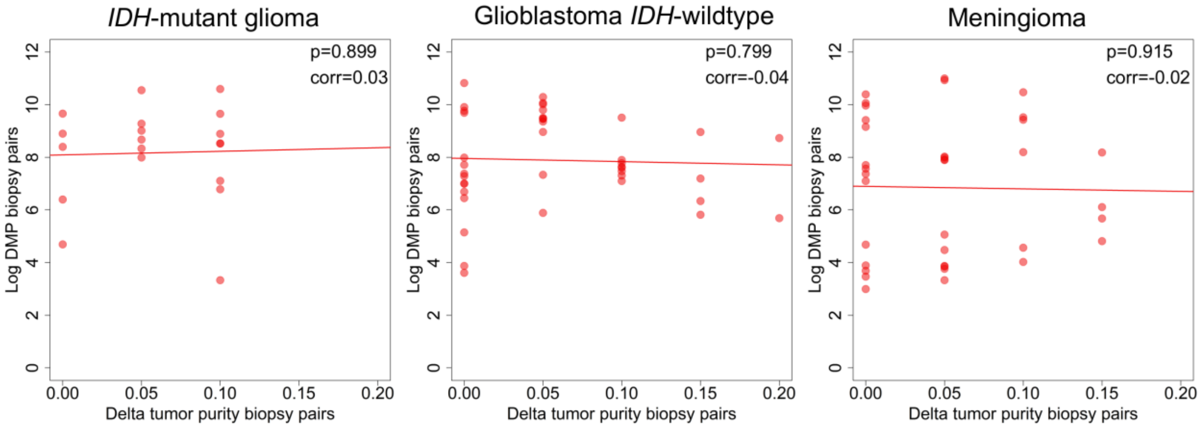

Supplement: Supplementary file 1 — Supplementary Figures [file 41379_2022_1113_MOESM1_ESM.pdf]
