# Supplementary material for: Spatial heterogeneity in DNA methylation and chromosomal alterations in diffuse gliomas and meningiomas
Source: Mod Pathol. 2022 Jun 14;35(11):1551–61. doi: 10.1038/s41379-022-01113-8 (PMC9596370; doi:10.1038/s41379-022-01113-8)
Supplement: Supplementary file 2 — Supplementary Table 1 [file 41379_2022_1113_MOESM2_ESM.pdf]

Sample_ID	DNA methylation superfamily (MNP v12.5)	DNA methylation superfamily calibrated score (MNP v12.5)	DNA methylation family (MNP v12.5)	DNA methylation family calibrated score (MNP v12.5)	DNA methylation family member (MNP v12.5)	DNA methylation family member calibrated score (MNP v12.5)
GU-LGG-72_1	Control tissues	0.99684	Control brain tissues	0.99681	Mc control tissue, cerebral hemisphere	0.99673
GU-LGG-72_2	Control tissues	0.99993	Control brain tissues	0.99993	Mc control tissue, cerebral hemisphere	0.99992
GU-LGG-72_3	Adult-type diffuse gliomas	0.78384	Diffuse glioma, IDH-mutant	0.77508	Mc oligodendroglioma, IDH-mutant and 1p/19q-codeleted	0.61668
GU-LGG-73_1	Adult-type diffuse gliomas	0.99502	Diffuse glioma, IDH-mutant	0.99478	Mc oligodendroglioma, IDH-mutant and 1p/19q-codeleted	0.97910
GU-LGG-73_2	Adult-type diffuse gliomas	0.99861	Diffuse glioma, IDH-mutant	0.99855	Mc oligodendroglioma, IDH-mutant and 1p/19q-codeleted	0.99629
GU-LGG-73_3	Adult-type diffuse gliomas	0.98745	Diffuse glioma, IDH-mutant	0.98671	Mc oligodendroglioma, IDH-mutant and 1p/19q-codeleted	0.82297
GU-LGG-83_1	Adult-type diffuse gliomas	0.99989	Diffuse glioma, IDH-mutant	0.99988	Mc oligodendroglioma, IDH-mutant and 1p/19q-codeleted	0.99984
GU-LGG-83_2	Adult-type diffuse gliomas	0.99989	Diffuse glioma, IDH-mutant	0.99999	Mc oligodendroglioma, IDH-mutant and 1p/19q-codeleted	0.99999
GU-LGG-83_3	Control tissues	0.99946	Control brain tissues	0.99946	Mc control tissue, cerebral hemisphere	0.99944
GU-LGG-83_4	Control tissues	0.98647	Control brain tissues	0.98638	Mc control tissue, cerebral hemisphere	0.98599
GU-LGG-88_4	Adult-type diffuse gliomas	0.99996	Diffuse glioma, IDH-mutant	0.99996	Mc oligodendroglioma, IDH-mutant and 1p/19q-codeleted	0.99972
GU-LGG-88_5	Adult-type diffuse gliomas	0.99998	Diffuse glioma, IDH-mutant	0.99998	Mc oligodendroglioma, IDH-mutant and 1p/19q-codeleted	0.99990
GU-LGG-88_6	Adult-type diffuse gliomas	0.99998	Diffuse glioma, IDH-mutant	0.99998	Mc oligodendroglioma, IDH-mutant and 1p/19q-codeleted	0.99994
GU-LGG-90_1	Adult-type diffuse gliomas	0.99961	Diffuse glioma, IDH-mutant	0.99960	Mc astrocytoma, IDH-mutant; lower-grade	0.97712
GU-LGG-90_2	Adult-type diffuse gliomas	0.99986	Diffuse glioma, IDH-mutant	0.99986	Mc astrocytoma, IDH-mutant; lower-grade	0.99451
GU-LGG-90_3	Adult-type diffuse gliomas	0.99919	Diffuse glioma, IDH-mutant	0.99917	Mc astrocytoma, IDH-mutant; lower-grade	0.84401
GU-LGG-91_1	Adult-type diffuse gliomas	0.99997	Diffuse glioma, IDH-mutant	0.99997	Mc astrocytoma, IDH-mutant; lower-grade	0.99786
GU-LGG-91_2	Adult-type diffuse gliomas	0.99997	Diffuse glioma, IDH-mutant	0.99996	Mc astrocytoma, IDH-mutant; lower-grade	0.99817
GU-LGG-91_3	Adult-type diffuse gliomas	0.99996	Diffuse glioma, IDH-mutant	0.99996	Mc astrocytoma, IDH-mutant; lower-grade	0.99592
GU-LGG-93_1	Adult-type diffuse gliomas	0.99974	Glioblastoma, IDH-wildtype	0.99972	Mc glioblastoma, IDH-wildtype, RTK2 subtype	0.99937
GU-LGG-93_2	Adult-type diffuse gliomas	0.94352	Glioblastoma, IDH-wildtype	0.93845	Mc glioblastoma, IDH-wildtype, mesenchymal subtype	0.85146
GU-LGG-93_3	Adult-type diffuse gliomas	0.50686	Glioblastoma, IDH-wildtype	0.41849	Mc glioblastoma, IDH-wildtype, mesenchymal subtype	0.34668
GU-LGG-96R_1	Adult-type diffuse gliomas	0.99975	Diffuse glioma, IDH-mutant	0.99974	Mc oligodendroglioma, IDH-mutant and 1p/19q-codeleted	0.99575
GU-LGG-96R_2	Adult-type diffuse gliomas	0.99989	Diffuse glioma, IDH-mutant	0.99989	Mc oligodendroglioma, IDH-mutant and 1p/19q-codeleted	0.99984
GU-LGG-96R_3	Adult-type diffuse gliomas	0.99991	Diffuse glioma, IDH-mutant	0.99991	Mc oligodendroglioma, IDH-mutant and 1p/19q-codeleted	0.99982
GU-LGG-98_1	Adult-type diffuse gliomas	0.99992	Diffuse glioma, IDH-mutant	0.99992	Mc astrocytoma, IDH-mutant; lower-grade	0.99937
GU-LGG-98_2	Control tissues	0.94405	Control brain tissues	0.94368	Mc control tissue, cerebral hemisphere	0.94249
GU-LGG-98_3	Adult-type diffuse gliomas	0.99999	Diffuse glioma, IDH-mutant	0.99999	Mc astrocytoma, IDH-mutant; lower-grade	0.99990
GU-LGG-99_1	Adult-type diffuse gliomas	0.99703	Diffuse glioma, IDH-mutant	0.99690	Mc oligodendroglioma, IDH-mutant and 1p/19q-codeleted	0.95372
GU-LGG-99_2	Adult-type diffuse gliomas	0.99992	Diffuse glioma, IDH-mutant	0.99992	Mc oligodendroglioma, IDH-mutant and 1p/19q-codeleted	0.99810
GU-LGG-99_3	Adult-type diffuse gliomas	0.98363	Diffuse glioma, IDH-mutant	0.98279	Mc oligodendroglioma, IDH-mutant and 1p/19q-codeleted	0.94338
GU-HGG-154_1	Adult-type diffuse gliomas	0.99910	Glioblastoma, IDH-wildtype	0.99905	Mc glioblastoma, IDH-wildtype, RTK2 subtype	0.99639
GU-HGG-154_2	Adult-type diffuse gliomas	0.99975	Glioblastoma, IDH-wildtype	0.99974	Mc glioblastoma, IDH-wildtype, RTK2 subtype	0.99947
GU-HGG-154_3	Adult-type diffuse gliomas	0.99987	Glioblastoma, IDH-wildtype	0.99986	Mc glioblastoma, IDH-wildtype, RTK2 subtype	0.99969
GU-HGG-154_4	Adult-type diffuse gliomas	0.99699	Glioblastoma, IDH-wildtype	0.99693	Mc glioblastoma, IDH-wildtype, RTK2 subtype	0.99585
GU-HGG-157_1	Low-grade glial/glioneuronal/neuroepithelial tumors	0.99195	Low-grade ganglioglial/neuroepithelial tumor	0.99106	Mc ganglioglioma	0.98804
GU-HGG-157_2	Low-grade glial/glioneuronal/neuroepithelial tumors	0.73381	Low-grade ganglioglial/neuroepithelial tumor	0.72942	Mc ganglioglioma	0.71786
GU-HGG-157_3	Adult-type diffuse gliomas	0.99822	Glioblastoma, IDH-wildtype	0.99820	Mc glioblastoma, IDH-wildtype, mesenchymal subtype	0.99812
GU-HGG-182_1	Adult-type diffuse gliomas	0.98954	Glioblastoma, IDH-wildtype	0.98896	Mc glioblastoma, IDH-wildtype, RTK2 subtype	0.64986
GU-HGG-182_2	Adult-type diffuse gliomas	0.92291	Glioblastoma, IDH-wildtype	0.92269	Mc glioblastoma, IDH-wildtype, mesenchymal subtype	0.91895
GU-HGG-182_3	Adult-type diffuse gliomas	0.99846	Glioblastoma, IDH-wildtype	0.99839	Mc glioblastoma, IDH-wildtype, RTK2 subtype	0.99147
GU-HGG-182_4	Adult-type diffuse gliomas	0.99315	Glioblastoma, IDH-wildtype	0.99283	Mc glioblastoma, IDH-wildtype, RTK2 subtype	0.94696
GU-HGG-185_1	Adult-type diffuse gliomas	1.00000	Glioblastoma, IDH-wildtype	0.99999	Mc glioblastoma, IDH-wildtype, RTK1 subtype	1.00000
GU-HGG-185_2	Adult-type diffuse gliomas	0.99999	Glioblastoma, IDH-wildtype	0.99999	Mc glioblastoma, IDH-wildtype, RTK1 subtype	0.99999
GU-HGG-185_3	Adult-type diffuse gliomas	0.99999	Glioblastoma, IDH-wildtype	0.99999	Mc glioblastoma, IDH-wildtype, RTK1 subtype	0.99999
GU-HGG-198_1	Adult-type diffuse gliomas	0.99999	Glioblastoma, IDH-wildtype	0.99999	Mc glioblastoma, IDH-wildtype, mesenchymal subtype	0.99999
GU-HGG-198_2	Adult-type diffuse gliomas	0.99984	Glioblastoma, IDH-wildtype	0.99982	Mc glioblastoma, IDH-wildtype, mesenchymal subtype	0.99867
GU-HGG-198_3	Control tissues	0.66190	Control brain tissues	0.64784	Mc control tissue, cerebral hemisphere	0.64059
GU-HGG-200_1	Adult-type diffuse gliomas	0.99989	Glioblastoma, IDH-wildtype	0.99989	Mc glioblastoma, IDH-wildtype, RTK2 subtype	0.99954
GU-HGG-200_2	Adult-type diffuse gliomas	0.99586	Glioblastoma, IDH-wildtype	0.99561	Mc glioblastoma, IDH-wildtype, RTK2 subtype	0.93746
GU-HGG-200_3	Adult-type diffuse gliomas	0.99974	Glioblastoma, IDH-wildtype	0.99972	Mc glioblastoma, IDH-wildtype, RTK2 subtype	0.99674
GU-HGG-204_1	Adult-type diffuse gliomas	0.99469	Glioblastoma, IDH-wildtype	0.99451	Mc glioblastoma, IDH-wildtype, mesenchymal subtype	0.98389
GU-HGG-204_2	Adult-type diffuse gliomas	0.99587	Glioblastoma, IDH-wildtype	0.99574	Mc glioblastoma, IDH-wildtype, RTK2 subtype	0.84792
GU-HGG-204_3	Adult-type diffuse gliomas	0.99999	Glioblastoma, IDH-wildtype	0.99999	Mc glioblastoma, IDH-wildtype, mesenchymal subtype	0.99998
GU-HGG-206_1	Adult-type diffuse gliomas	0.97629	Glioblastoma, IDH-wildtype	0.97517	Mc glioblastoma, IDH-wildtype, mesenchymal subtype	0.52254
GU-HGG-206_2	Adult-type diffuse gliomas	0.92139	Glioblastoma, IDH-wildtype	0.91132	Mc glioblastoma, IDH-wildtype, mesenchymal subtype	0.90976
GU-HGG-206_3	Adult-type diffuse gliomas	0.99741	Glioblastoma, IDH-wildtype	0.99731	Mc glioblastoma, IDH-wildtype, RTK2 subtype	0.98845
GU-HGG-216_1	Adult-type diffuse gliomas	0.99991	Diffuse glioma, IDH-mutant	0.99990	Mc astrocytoma, IDH-mutant; high-grade	0.99795
GU-HGG-216_2	Adult-type diffuse gliomas	0.99944	Diffuse glioma, IDH-mutant	0.99942	Mc astrocytoma, IDH-mutant; high-grade	0.93761
GU-HGG-216_3	Adult-type diffuse gliomas	0.99984	Diffuse glioma, IDH-mutant	0.99983	Mc astrocytoma, IDH-mutant; high-grade	0.99292
GU-HGG-224_1	Adult-type diffuse gliomas	0.33671	Glioblastoma, IDH-wildtype	0.32178	Mc glioblastoma, IDH-wildtype, mesenchymal subtype	0.21166
GU-HGG-224_2	Adult-type diffuse gliomas	0.98019	Glioblastoma, IDH-wildtype	0.97947	Mc glioblastoma, IDH-wildtype, mesenchymal subtype	0.96667
GU-HGG-224_3	Adult-type diffuse gliomas	0.56929	Glioblastoma, IDH-wildtype	0.55841	Mc glioblastoma, IDH-wildtype, mesenchymal subtype	0.49189
GU-HGG-225_1	Adult-type diffuse gliomas	0.99999	Glioblastoma, IDH-wildtype	0.99999	Mc glioblastoma, IDH-wildtype, RTK1 subtype	0.99999
GU-HGG-225_2	Adult-type diffuse gliomas	0.99999	Glioblastoma, IDH-wildtype	0.99999	Mc glioblastoma, IDH-wildtype, RTK1 subtype	0.99999
GU-HGG-225_3	Adult-type diffuse gliomas	0.99999	Glioblastoma, IDH-wildtype	0.99999	Mc glioblastoma, IDH-wildtype, RTK1 subtype	0.99999
GU-HGG-260_1	Pediatric-type diffuse high-grade gliomas	0.85137	Diffuse pediatric-type high-grade glioma, H3-\	0.84884	Mc diffuse pediatric-type high grade glioma, RTK1 subtype, subclass c (novel)	0.83516
GU-HGG-260_2	Pediatric-type diffuse high-grade gliomas	0.87830	Diffuse pediatric-type high-grade glioma, H3-\	0.87685	Mc diffuse pediatric-type high grade glioma, RTK1 subtype, subclass c (novel)	0.86725
GU-HGG-260_3	Pediatric-type diffuse high-grade gliomas	0.83755	Diffuse pediatric-type high-grade glioma, H3-\	0.83536	Mc diffuse pediatric-type high grade glioma, RTK1 subtype, subclass c (novel)	0.82341
GU-HGG-269_1	Adult-type diffuse gliomas	0.99945	Glioblastoma, IDH-wildtype	0.99941	Mc glioblastoma, IDH-wildtype, RTK1 subtype	0.90944
GU-HGG-269_2	Adult-type diffuse gliomas	0.99978	Glioblastoma, IDH-wildtype	0.99977	Mc glioblastoma, IDH-wildtype, RTK1 subtype	0.98215
GU-HGG-269_3	Adult-type diffuse gliomas	0.99981	Glioblastoma, IDH-wildtype	0.99980	Mc glioblastoma, IDH-wildtype, RTK1 subtype	0.96833
GU-HGG-271_1	Adult-type diffuse gliomas	0.97455	Glioblastoma, IDH-wildtype	0.97368	Mc glioblastoma, IDH-wildtype, RTK2 subtype	0.73215
GU-HGG-271_2	Adult-type diffuse gliomas	0.99994	Glioblastoma, IDH-wildtype	0.99994	Mc glioblastoma, IDH-wildtype, RTK1 subtype	0.99971
GU-HGG-271_3	Adult-type diffuse gliomas	0.99225	Glioblastoma, IDH-wildtype	0.99194	Mc glioblastoma, IDH-wildtype, mesenchymal subtype	0.95047
GU-HGG-287_1	Adult-type diffuse gliomas	0.86860	Glioblastoma, IDH-wildtype	0.86638	Mc glioblastoma, IDH-wildtype, RTK2 subtype	0.58955
GU-HGG-287_2	Adult-type diffuse gliomas	0.99989	Glioblastoma, IDH-wildtype	0.99986	Mc glioblastoma, IDH-wildtype, mesenchymal subtype	0.99950
GU-HGG-287_3	Adult-type diffuse gliomas	0.99909	Glioblastoma, IDH-wildtype	0.99898	Mc glioblastoma, IDH-wildtype, mesenchymal subtype	0.99481
GU-HGG-287_4	Adult-type diffuse gliomas	0.99975	Glioblastoma, IDH-wildtype	0.99972	Mc glioblastoma, IDH-wildtype, mesenchymal subtype	0.99914
GU-HGG-287_5	Adult-type diffuse gliomas	0.99961	Glioblastoma, IDH-wildtype	0.99957	Mc glioblastoma, IDH-wildtype, mesenchymal subtype	0.99814
GU-HGG-364_1	Adult-type diffuse gliomas	0.39618	Glioblastoma, IDH-wildtype	0.34724	Mc glioblastoma, IDH-wildtype, mesenchymal subtype	0.32898
GU-HGG-364_2	Control tissues	0.96034	Control brain tissues	0.95937	Mc control tissue, cerebral hemisphere	0.95849
GU-HGG-364_3	Adult-type diffuse gliomas	0.99995	Glioblastoma, IDH-wildtype	0.99994	Mc glioblastoma, IDH-wildtype, mesenchymal subtype	0.99981
GU-HGG-365_1	Adult-type diffuse gliomas	0.97419	Diffuse glioma, IDH-mutant	0.96972	Mc astrocytoma, IDH-mutant; high-grade	0.77761
GU-HGG-365_2	Adult-type diffuse gliomas	0.98757	Diffuse glioma, IDH-mutant	0.98622	Mc astrocytoma, IDH-mutant; lower-grade	0.81585
GU-HGG-365_3	Adult-type diffuse gliomas	0.84165	Diffuse glioma, IDH-mutant	0.82849	Mc astrocytoma, IDH-mutant; lower-grade	0.47681
GU-HGG-365_4	Adult-type diffuse gliomas	0.99266	Diffuse glioma, IDH-mutant	0.99207	Mc astrocytoma, IDH-mutant; lower-grade	0.88899
GU-HGG-365_5	Adult-type diffuse gliomas	0.99020	Diffuse glioma, IDH-mutant	0.98942	Mc astrocytoma, IDH-mutant; lower-grade	0.81827
GU-lgMNG-1_1	Meningioma	0.99997	Meningioma	0.99997	Mc meningioma, subtype benign, subclass 1 (novel)	0.99990
GU-lgMNG-1_2	Meningioma	0.99995	Meningioma	0.99995	Mc meningioma, subtype benign, subclass 1 (novel)	0.99983
GU-lgMNG-1_3	Meningioma	0.99996	Meningioma	0.99996	Mc meningioma, subtype benign, subclass 1 (novel)	0.99985
GU-lgMNG-2_1	Meningioma	0.99978	Meningioma	0.99978	Mc meningioma, subtype intermediate, subclass a (novel)	0.94316
GU-lgMNG-2_2	Meningioma	0.99840	Meningioma	0.99840	Mc meningioma, subtype benign, subclass 2 (novel)	0.50552
GU-lgMNG-2_3	Meningioma	0.99963	Meningioma	0.99963	Mc meningioma, subtype intermediate, subclass a (novel)	0.91163
GU-lgMNG-3_1	Meningioma	0.99999	Meningioma	0.99999	Mc meningioma, subtype benign, subclass 1 (novel)	0.99999
GU-lgMNG-3_2	Meningioma	0.99999	Meningioma	0.99999	Mc meningioma, subtype benign, subclass 1 (novel)	0.99999
GU-lgMNG-3_3	Meningioma	0.99999	Meningioma	0.99999	Mc meningioma, subtype benign, subclass 1 (novel)	0.99999
GU-hgMNG-7_1	Meningioma	0.99958	Meningioma	0.99958	Mc meningioma, subtype benign, subclass 1 (novel)	0.99733
GU-hgMNG-7_2	Meningioma	0.99756	Meningioma	0.99756	Mc meningioma, subtype benign, subclass 1 (novel)	0.91356
GU-hgMNG-7_3	Meningioma	0.99924	Meningioma	0.99924	Mc meningioma, subtype benign, subclass 1 (novel)	0.99000
GU-hgMNG-8R_1	Meningioma	0.99303	Meningioma	0.99303	Mc meningioma, subtype benign, subclass 3 (novel)	0.68770
GU-hgMNG-8R_2	Meningioma	0.99603	Meningioma	0.99603	Mc meningioma, subtype benign, subclass 3 (novel)	0.84006
GU-hgMNG-8R_3	Meningioma	0.99484	Meningioma	0.99484	Mc meningioma, subtype benign, subclass 3 (novel)	0.76119
GU-hgMNG-8R_4	Meningioma	0.99388	Meningioma	0.99388	Mc meningioma, subtype benign, subclass 3 (novel)	0.79736
GU-hgMNG-13R_1	Meningioma	0.98404	Meningioma	0.98404	Mc meningioma, subtype intermediate, subclass a (novel)	0.95266
GU-hgMNG-13R_2	Meningioma	0.99998	Meningioma	0.99998	Mc meningioma, subtype intermediate, subclass b (novel)	0.78147
GU-hgMNG-13R_3	Meningioma	0.99999	Meningioma	0.99999	Mc meningioma, subtype malignant (novel)	0.99215
GU-hgMNG-14_1	Meningioma	0.99885	Meningioma	0.99885	Mc meningioma, subtype benign, subclass 1 (novel)	0.85870
GU-hgMNG-14_2	Meningioma	0.99876	Meningioma	0.99876	Mc meningioma, subtype intermediate, subclass a (novel)	0.81496
GU-hgMNG-14_3	Meningioma	0.99903	Meningioma	0.99903	Mc meningioma, subtype intermediate, subclass a (novel)	0.96410
GU-hgMNG-14_4	Meningioma	0.99999	Meningioma	0.99999	Mc meningioma, subtype intermediate, subclass b (novel)	0.99998
GU-hgMNG-14R_1	Meningioma	0.99628	Meningioma	0.99628	Mc meningioma, subtype malignant (novel)	0.93406
GU-hgMNG-14R_2	Meningioma	0.99756	Meningioma	0.99756	Mc meningioma, subtype malignant (novel)	0.93409
GU-hgMNG-14R_3	Meningioma	0.99735	Meningioma	0.99735	Mc meningioma, subtype intermediate, subclass b (novel)	0.70575
GU-hgMNG-16_1	Meningioma	0.99969	Meningioma	0.99969	Mc meningioma, subtype intermediate, subclass a (novel)	0.99630
GU-hgMNG-16_2	Meningioma	0.99835	Meningioma	0.99835	Mc meningioma, subtype intermediate, subclass a (novel)	0.86304
GU-hgMNG-16_3	Meningioma	0.99921	Meningioma	0.99921	Mc meningioma, subtype intermediate, subclass a (novel)	0.98666
GU-hgMNG-18_1	Meningioma	0.99999	Meningioma	0.99999	Mc meningioma, subtype benign, subclass 2 (novel)	0.99813
GU-hgMNG-18_2	Meningioma	0.99999	Meningioma	0.99999	Mc meningioma, subtype benign, subclass 2 (novel)	0.99939
GU-hgMNG-18_3	Meningioma	0.99999	Meningioma	0.99999	Mc meningioma, subtype benign, subclass 2 (novel)	0.99968
GU-hgMNG-20_1	Meningioma	0.99967	Meningioma	0.99967	Mc meningioma, subtype intermediate, subclass a (novel)	0.99823
GU-hgMNG-20_2	Meningioma	0.99992	Meningioma	0.99992	Mc meningioma, subtype intermediate, subclass a (novel)	0.99752
GU-hgMNG-20_3	Meningioma	0.99676	Meningioma	0.99676	Mc meningioma, subtype intermediate, subclass a (novel)	0.90962
GU-hgMNG-23R_1	Meningioma	0.99812	Meningioma	0.99812	Mc meningioma, subtype benign, subclass 3 (novel)	0.98787
GU-hgMNG-23R_2	Meningioma	0.99997	Meningioma	0.99997	Mc meningioma, subtype benign, subclass 3 (novel)	0.99913
GU-hgMNG-23R_3	Meningioma	0.99973	Meningioma	0.99973	Mc meningioma, subtype benign, subclass 3 (novel)	0.99536

MNP: Molecular neuropathology brain classifier, version 12.5
GU-LGG: lower-grade glioma
GU-HGG: high-grade glioma
GU-lgMNG: low-grade MNG
GU-hgMNG: high-grade MNG
R: Recurrent tumor
