# Supplementary material for: Spatial heterogeneity in DNA methylation and chromosomal alterations in diffuse gliomas and meningiomas
Source: Mod Pathol. 2022 Jun 14;35(11):1551–61. doi: 10.1038/s41379-022-01113-8 (PMC9596370; doi:10.1038/s41379-022-01113-8)
Supplement: Supplementary file 3 — Supplementary Table 2 [file 41379_2022_1113_MOESM3_ESM.pdf]

Sample_ID	Number of biopsies	Diagnosis according to 2016 WHO (Louis DN et al. 2016)	Reclassification according to 2021 WHO (Louis DN et al. 2021)
GU-LGG-88	3	Diffuse oligodendroglioma, *IDH* -mutant and 1p/19q codeleted, WHO (2016) grade II	Oligodendroglioma, *IDH* -mutant and 1p/19q-codeleted, CNS WHO grade 2
GU-LGG-99	3	Diffuse oligodendroglioma, *IDH* -mutant and 1p/19q codeleted, WHO (2016) grade II	Oligodendroglioma, *IDH* -mutant and 1p/19q-codeleted, CNS WHO grade 2
GU-LGG-72	3	Anaplastic oligodendroglioma, *IDH* -mutant and 1p/19q codeleted, WHO (2016) grade III	Oligodendroglioma, *IDH* -mutant and 1p/19q-codeleted, CNS WHO grade 3
GU-LGG-73	3	Anaplastic oligodendroglioma, *IDH* -mutant and 1p/19q codeleted, WHO (2016) grade III	Oligodendroglioma, *IDH* -mutant and 1p/19q-codeleted, CNS WHO grade 3
GU-LGG-83R	4	Anaplastic oligodendroglioma, *IDH* -mutant and 1p/19q codeleted, WHO (2016) grade III	Oligodendroglioma, *IDH* -mutant and 1p/19q-codeleted, CNS WHO grade 3
GU-LGG-96R	3	Anaplastic oligodendroglioma, *IDH* -mutant and 1p/19q codeleted, WHO (2016) grade III	Oligodendroglioma, *IDH* -mutant and 1p/19q-codeleted, CNS WHO grade 3
GU-LGG-90	3	Diffuse astrocytoma, *IDH* -mutant, WHO (2016) grade II	Astrocytoma, *IDH* -mutant, CNS WHO grade 4*
GU-LGG-91	3	Diffuse astrocytoma, *IDH* -mutant, WHO (2016) grade II	Astrocytoma, *IDH* -mutant, CNS WHO grade 2
GU-LGG-98	3	Diffuse astrocytoma, *IDH* -mutant, WHO (2016) grade II	Astrocytoma, *IDH* -mutant, CNS WHO grade 2
GU-LGG-93	3	Anaplastic astrocytoma, *IDH* -wildtype, WHO (2016) grade III	Glioblastoma, *IDH* -wildtype, CNS WHO grade 4
GU-HGG-154†	4	Glioblastoma, *IDH* -wildtype, WHO (2016) grade IV	Glioblastoma, *IDH* -wildtype, CNS WHO grade 4
GU-HGG-157†	3	Glioblastoma, *IDH* -wildtype, WHO (2016) grade IV	Glioblastoma, *IDH* -wildtype, CNS WHO grade 4
GU-HGG-182†	4	Glioblastoma, *IDH* -wildtype, WHO (2016) grade IV	Glioblastoma, *IDH* -wildtype, CNS WHO grade 4
GU-HGG-185†	3	Glioblastoma, *IDH* -wildtype, WHO (2016) grade IV	Glioblastoma, *IDH* -wildtype, CNS WHO grade 4
GU-HGG-198†	3	Glioblastoma, *IDH* -wildtype, WHO (2016) grade IV	Glioblastoma, *IDH* -wildtype, CNS WHO grade 4
GU-HGG-200†	3	Glioblastoma, *IDH* -wildtype, WHO (2016) grade IV	Glioblastoma, *IDH* -wildtype, CNS WHO grade 4
GU-HGG-204†	3	Glioblastoma, *IDH* -wildtype, WHO (2016) grade IV	Glioblastoma, *IDH* -wildtype, CNS WHO grade 4
GU-HGG-206†	3	Glioblastoma, *IDH* -wildtype, WHO (2016) grade IV	Glioblastoma, *IDH* -wildtype, CNS WHO grade 4
GU-HGG-224†	3	Glioblastoma, *IDH* -wildtype, WHO (2016) grade IV	Glioblastoma, *IDH* -wildtype, CNS WHO grade 4
GU-HGG-225†	3	Glioblastoma, *IDH* -wildtype, WHO (2016) grade IV	Glioblastoma, *IDH* -wildtype, CNS WHO grade 4
GU-HGG-260†	3	Glioblastoma, *IDH* -wildtype, WHO (2016) grade IV	Glioblastoma, *IDH* -wildtype, CNS WHO grade 4
GU-HGG-269†	3	Glioblastoma, *IDH* -wildtype, WHO (2016) grade IV	Glioblastoma, *IDH* -wildtype, CNS WHO grade 4
GU-HGG-271†	3	Glioblastoma, *IDH* -wildtype, WHO (2016) grade IV	Glioblastoma, *IDH* -wildtype, CNS WHO grade 4
GU-HGG-287†	5	Glioblastoma, *IDH* -wildtype, WHO (2016) grade IV	Glioblastoma, *IDH* -wildtype, CNS WHO grade 4
GU-HGG-364	3	Glioblastoma, *IDH* -wildtype, WHO (2016) grade IV	Glioblastoma, *IDH* -wildtype, CNS WHO grade 4
GU-HGG-216†	3	Glioblastoma, *IDH* -mutant, WHO (2016) grade IV	Astrocytoma, *IDH* -mutant, CNS WHO grade 4
GU-HGG-365	5	Glioblastoma, *IDH* -mutant, WHO (2016) grade IV	Astrocytoma, *IDH* -mutant, CNS WHO grade 4
GU-lgMNG-1†	3	Meningioma, WHO (2016) grade I	Meningioma, CNS WHO grade 1
GU-lgMNG-2†	3	Meningioma, WHO (2016) grade I	Meningioma, CNS WHO grade 1
GU-lgMNG-3†	3	Meningioma, WHO (2016) grade I	Meningioma, CNS WHO grade 1
GU-hgMNG-7	3	Meningioma, WHO (2016) grade II	Meningioma, CNS WHO grade 2
GU-hgMNG-8R	4	Meningioma, WHO (2016) grade II	Meningioma, CNS WHO grade 2
GU-hgMNG-13R	3	Meningioma, WHO (2016) grade II	Meningioma, CNS WHO grade 2
GU-hgMNG-14	4	Meningioma, WHO (2016) grade II**	Meningioma, CNS WHO grade 2***
GU-hgMNG-14R	3	Meningioma, WHO (2016) grade II	Meningioma, CNS WHO grade 2
GU-hgMNG-16	3	Meningioma, WHO (2016) grade II	Meningioma, CNS WHO grade 2
GU-hgMNG-18	3	Meningioma, WHO (2016) grade II	Meningioma, CNS WHO grade 2
GU-hgMNG-20	3	Meningioma, WHO (2016) grade II	Meningioma, CNS WHO grade 2
GU-hgMNG-23R	3	Meningioma, WHO (2016) grade II	Meningioma, CNS WHO grade 2

GU-LGG: lower-grade glioma
GU-HGG: high-grade glioma
GU-lgMNG: low-grade MNG
GU-hgMNG: high-grade MNG
R: Recurrent tumor
*Tumor regions of CNS WHO grade 2 and 4
**Tumor regions of WHO (2016) grade I and II
***Tumor regions of CNS WHO grade 1 and 2
†From Wenger et al. 2019
